# Retinoid Therapy in a Case of Harlequin Ichthyosis with a Short Literature Review

**DOI:** 10.1155/2024/8729318

**Published:** 2024-01-12

**Authors:** Emad Bahashwan, Jaber Alfaifi, Sahar Elmaghawri Mohamed Moursi, Youssef Elbayoumi Soliman

**Affiliations:** ^1^Division of Dermatology, Department of Internal Medicine, College of Medicine, University of Bisha, Bisha, Saudi Arabia; ^2^Department of Child Health, College of Medicine, University of Bisha, Bisha, Saudi Arabia; ^3^Maternal Child Hospital, Bisha, Saudi Arabia; ^4^Dermatology, Andrology and STDs, Faculty of Medicine, Mansoura University, Mansoura, Egypt

## Abstract

Harlequin ichthyosis (HI) is a genetically inherited epidermal disorder due to the mutation of the ABCA12 gene, which is responsible for lipid transportation, and presents with large keratinised scales characterised by deep erythematous fissures, with ectropion and eclabium. A moderate number of cases and a high mortality rate have been recorded. In this case report, a pregnant lady gave birth to a 33-week-old premature foetus with characteristic symptoms of HI. After admitting him to the NICU, a multidisciplinary treatment approach was conducted with paediatric dermatologists, ophthalmologists, urologists, and dieticians. The prognosis is positive, with desquamation of the hyperkeratotic plate revealing an erythematous and shiny skin. A short literature review on HI characteristics, diagnostic aids, and management has also been added.

## 1. Introduction

Harlequin ichthyosis (HI) is one of the rarest and most extreme conditions of genetically acquired ichthyosis. The neonates born with this disease are encased in a thick, dense, large hyperkeratotic, armour-like covering, with scattered, deep, and erythematous fissures at birth. The condition may additionally present with dysplastic ears and nose, ectropion of both eyes and eclabium (eversion of lips). It has been observed that this covering resists the movement of neonates' limbs [1]. Its incidence is approximately 1 in 300,000 neonates. Reverend Oliver Hart, in 1750, reported its first case. The first line of defence, i.e., skin, is disrupted, and microorganisms, including infectious bacteria, could easily reach the sublying organs and tissues. The major reason for early death mostly includes hypothermia, respiratory distress, septicaemia, and complications due to dehydration [2, 3].

**Figure 1:**
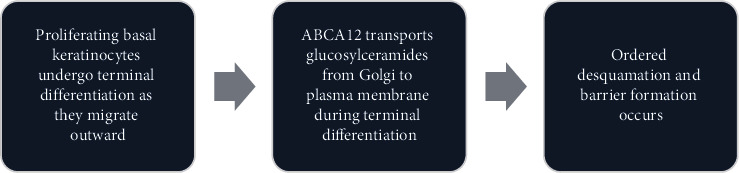
Normal keratinocyte differentiation and lipid transport.

Earlier, the pathophysiology of Harlequin ichthyosis is not revealed. Akiyama et al. [4] revealed that this genetic disorder is due to the mutation of a gene responsible for lipid metabolism. The pathogenesis of Harlequin ichthyosis results from mutations in the ABCA12 gene which encodes an ATP-binding cassette (ABC) transporter protein also known as ABCA12 [5, 6]. ABCA12 plays an essential role in keratinocyte differentiation through transporting glucosylceramides from the Golgi apparatus to the plasma membrane (Figure 1). In the normal epidermal differentiation process (Figure 1), proliferating basal keratinocytes undergo terminal differentiation as they migrate outward. This involves the precise regulation of lipid composition and transport mediated by proteins such as ABCA12 [4].

**Figure 2:**
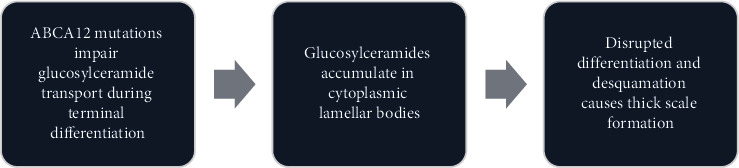
Pathogenesis in Harlequin ichthyosis.

Mutations in ABCA12 impair its function, resulting in defective transport of glucosylceramides during terminal differentiation [4] (Figure 2). Without ABCA12 activity, glucosylceramides accumulate in the cytoplasmic lamellar bodies instead of being exported to the extracellular space. The intracellular accumulation of glucosylceramides disturbs the orderly desquamation process and causes extremely thickened plaques of hyperkeratotic scale to form over the entire skin surface, a hallmark of Harlequin ichthyosis [4].

Within these scales, keratinocytes exhibit abnormal differentiation with disrupted intracellular lamellar membranes and organelles. The altered ABCA12 gene produces abnormal and nonfunctional proteins, imparting lipid transport and resulting in defective skin development, including increased keratin production and desquamation of the skin [7, 8]. The mortality rate of Harlequin ichthyosis was high due to a lack of research and the availability of resources. Enhanced critical management of the patient with initial treatment with systemic retinoid would increase the chances of survival [6]. Acitretin is a second-generation retinoid commonly used to treat Harlequin ichthyosis [9]. As a ligand for retinoic acid receptors (RARs), acitretin activates intracellular retinoid signaling pathways [9]. It has high affinity for RAR*γ* which is predominantly expressed in epidermis [10, 11]. RAR*γ* signaling modulates the expression of genes related to keratinocyte proliferation, differentiation, and desquamation [12]. Through its action on RAR*γ*, acitretin is able to partially compensate for the lipid transport defect in Harlequin ichthyosis by indirectly restoring a more normal epidermal structure [12]. Specifically, it enhances keratinocyte differentiation, facilitates lipid transport, and improves desquamation despite the underlying ABCA12 mutations [12].

Harlequin ichthyosis is an autosomal recessive congenital trait. The homozygous mutations of ABCA12 block the production of ABCA12 protein, eventually resulting in the severe phenotype of this disease. In contrast, the heterozygous substitution of the amino leads to a less severe phenotype [13]. Here, we present a case report of a premature neonate with typical characteristics of Harlequin ichthyosis treated with a multidisciplinary perspective, including dermatologists, urologists, and ophthalmologists. This report could guide the future of treating these special and rare patients.

## 2. Case Presentation

**Figure 3:**
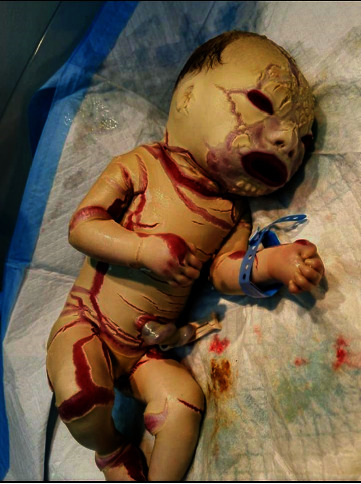
At birth.

This is a case of a male baby having 2.2 kg weight, delivered normally through the vagina at the 33^rd^ week of gestation. The mother was admitted to the hospital with no comorbidities, family history, and consanguinity and received a full dose of dexamethasone. The APGAR score was obtained by evaluating the newborn's health status at 8 and 9 on 1 and 5 minutes, respectively. Analysis of the cord blood gases was completed. pH is 7.33, PCO_2_ was 37.1 mmHg, and HCO_3_ was 19.5 mmol/L. The patient was shifted to NICU and placed in a humidified incubator. The infant had a heart rate of 130 beats per minute, a blood pressure of 67/43 mmHg, an oxygen saturation of 96%, and an average respiratory rate of 40 breaths per minute. At delivery, the infant was covered with a hyperkeratotic hard and thick skin extending to arms and extremities in diamond-shaped plates separated by deep erythematous fissures. Distortion of the lips (eclabium) and upper eyelid eversion (ectropion) were also observed (Figure 3).

The patient was started on oxygen therapy of 3 L/minute at 30% and a humidity of 80%. Upon examination, the central nervous system showed poor activity with no convulsions, and the cardiovascular system was normal with no murmur sounds. In the abdomen, there were audible intestinal sounds. Also, a small part of the penis was present with hard skin covering the scrotum, and the testes were not palpable. Ten percent dextrose was started at 60 mL/kg daily, with antibiotics including ampicillin, gentamycin, and fentanyl every 4 hours. Paraffin oil as a topical emollient and erythromycin eye ointment were advised after consultation with dermatologists and ophthalmologists, respectively. From the second day of delivery, acitretin 0.5 mg/kg daily was adminitered orally. Complete blood count and chemical tests for cord blood gases were repeated on day 2, showing the same results.

Additionally, the whole exome sequence (WES) test later confirmed the pathogenic variant in the mutated ABCA12 gene. Orogastric feeding of 3 cc for 3 hours was started with an OG tube on the second day. After the fifth day of delivery, sepsis and apnea were observed. The patient suffers from bradycardia with a heart rate of about 60 beats per minute and oxygen saturation (SpO_2_) decreased to 50%. According to the complete blood picture (CP), haemoglobin was 15 g/dL, white blood cells were 6 × 10^9^/L, and platelets were 90 × 10^9^/L. Investigations of sepsis from the laboratory revealed an *Escherichia coli* infection, so oxacillin and amikacin were started along with the intubation of the patient.

The lab results were normal, which means that the baby did not have any major medical problems at the time of discharge, as shown in Table 1. The lab results of the Harlequin ichthyosis baby at the time of discharge showed that the baby's white blood cell count (WBC) was slightly elevated, but still within the normal range. The baby's neutrophil percentage was also within the normal range. Red blood cell count (RBC), hemoglobin (HGB), hematocrit (HCT), mean corpuscular volume (MCV), mean corpuscular hemoglobin (MCH), mean corpuscular hemoglobin concentration (MCHC), red blood cell distribution width (RDW), and RDW standard deviation (RDW-SD) were all within the normal range. Platelet count (PLT) is slightly elevated, but still within the normal range. Mean platelet volume (MPV) is also within the normal range. These results suggested that the baby was generally healthy and did not have any major infections or other medical conditions. However, the slightly elevated WBC and platelet counts may have been a sign of a mild infection or other inflammatory condition.

**Figure 4:**
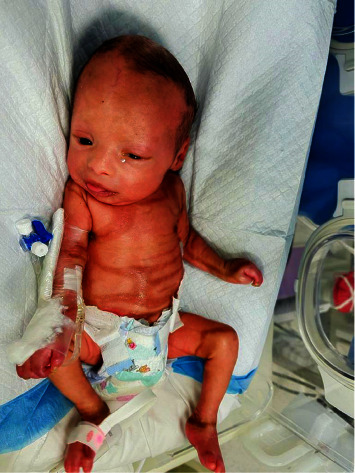
At 2 months old.

Extubation was done after 3 days, and the patient was placed on a ventilator, gradually replaced by oxygen therapy until the patient could breathe in room air. Feeding increased moderately till the patient was fully capable of ingesting orally. There was a progressive desquamation of hyperkeratinized plates with improved ectropion and eclabium, after two months of treatment (Figure 4). After 3^rd^ week, dermatologists lowered the dose of acitretin to 0.25 mg/kg daily. The patient was discharged after 30 days. Topical emollients and systemic acitretin were continued and dermatology follow-up was advised. The genetic diagnosis of autosomal recessive Harlequin congenital ichthyosis was confirmed by the whole exome sequence (WES) test mentioned above.

## 3. Discussion

Harlequin ichthyosis (HI) is a rare and extremely severe type of congenital ichthyosis with a distinctive and characteristic appearance at birth. It is also called as “Ichthyosis congenita” or “Keratosis diffusa foetalis”. According to the scientific literature reports in 2007, more or less 101 medical case reports regarding HI are present worldwide [14–16]. It is believed to be caused by a pathogenic variant of the ABCA12 gene, which is responsible for intracellular lipid transport. Defective lipid production leads to the deterioration of the skin lipid barrier between epidermal keratinocytes and assists in developing Harlequin ichthyosis [3, 4].

Based on the appearance of the patient at birth, a high index of clinical suspicion may prompt the provider to conduct genetic testing to confirm the diagnosis of HI. In the prenatal period, the diagnosis of HI can be confirmed by chorionic villus or amniotic fluid samples after discovering the ABCA12 gene mutation with a family history. Skin biopsy, fetoscopy, and microscopic examination of hair cells could also be helpful in patients prenatally for DNA analysis [17, 18]. The distinctive attributes of HI remained undetectable in ultrasound until they began to hinder the infant's development, typically in the third trimester. It is evident after an extensive literature review that only a few cases had been diagnosed by prenatal ultrasound. Sonography is also used for the diagnosis, but it cannot differentiate HI from other fetal diseases, including fetal macroglossia, which is also present in patients with Down's syndrome (Trisomy 21). Therefore, DNA sampling is the standard procedure to differentiate macroglossia from HI. Amniocentesis and Chorionic villus sampling could be done after 15 weeks and 10 weeks, respectively, but the latter has a slightly higher rate of miscarriage [19–21].

Initial management for HI begins with placing the infant in a humidified incubator for temperature regulation, preventing dehydration and microbial invasion, and maintaining homeostasis. The incubator is necessary to balance fluid, electrolytes, and heat loss due to abnormal functioning of the epidermal permeability barrier. It is also important to observe the infant's vitals and breathing pattern as respiration and circulation of the body could be compromised by the hyperkeratotic skin. According to studies, HI neonates require 25% extra calories daily than other infants [3, 22, 23]. Topical emollients may help discard the collodion membrane and assist in decreasing the hyperkeratotic plate. Bathing is necessary for these neonates to lower the risk of infection, soften the thick stratum corneum, and speed up the desquamation of the skin [24].

Oral retinoids such as acitretin have been instrumental in the treatment of severe inherited ichthyoses like Harlequin ichthyosis [25]. The primary advantages are their efficacy in improving epidermal structure and barrier function [25]. Several case reports have documented acitretin's clinical benefits in Harlequin ichthyosis [26, 27]. Early initiation of high-dose acitretin (0.8–1 mg/kg/day) resulted in decreased scaling and improved survival in neonates [27]. Long-term use maintained remission of skin manifestations and allowed patients to survive well beyond the newborn period when untreated mortality is extremely high [26]. However, oral retinoid therapy requires careful monitoring and management of potential side effects. Mild side effects like cheilitis, dryness, and musculoskeletal pains are common [27]. More serious adverse reactions can also occur such as hyperlipidemia, hepatotoxicity, and skeletal abnormalities with prolonged use [27]. Teratogenicity is a major concern, necessitating effective contraception during treatment in adult females. Dosing must be individualized based on clinical response and tolerability [27]. The survival rate of the neonate depends upon neonatal care and prescribed oral retinoid therapy. Several retinoids are used in the treatment of HI including isotretinoin, etretinate, and acitretin, with acitretin being the most used retinoid due to its preferred, moderate half-life and better safety profile. It accelerates the shedding of hyperkeratotic skin plates and improves ectropion and eclabium. According to a study conducted by Rajpopat et al., there is an 83% survival rate in an acitretin-treated group of 20 patients as compared to the 73% success rate in a control group [1, 8, 28, 29]. Patients exhibiting the condition with ectropion should be referred to an ophthalmologist. Antibiotics and antifungals could be started initially with surgical correction in infancy, as observed in previous reports [19].

## 4. Conclusion

Harlequin ichthyosis is a rare and challenging disorder, even in this era. Its mortality rate is high, and many neonates die due to severe complications, including dehydration, respiratory distress, and systemic infections. However, it is possible to overcome these complications with the help of a multidisciplinary approach to better optimise patient outcomes. Limitations include side effects of excessive use of retinoids which could cause heavier damage to the systemic organs in future. However, more research is warranted to provide safer treatment options with fewer postoperative complications in patients with HI. To conclude, oral retinoids with topical emollients remain the first-line treatment for patients with HI.

## Figures and Tables

**Table 1:** Lab results at the time of discharge.

Test	Results	Flags	Units	Low	High
WBC	14.17	Rh	×10^3^/*μ*L	4.00	11.00
LY	46.96	Rh	%	15.20	43.30
MO	11.35	R	%	5.50	13.70
NE	38.44	Rl	%	43.50	73.50
EO	2.67	R	%	0.80	8.10
BA	0.57	R	%	0.20	1.50
LY#	6.65	RH	×10^3^/*μ*L	1.20	4.00
MO#	1.01	RH	×10^3^/*μ*L	0.30	1.00
NE#	5.45	R	×10^3^/*μ*L	1.70	7.00
EO#	0.38	R	×10^3^/*μ*L	0.00	0.50
BA#	0.08	R	×10^3^/*μ*L	0.00	0.10
RBC	3.45	I	×10^4^/*μ*L	4.06	5.63
HGB	11.07	I	g/dL	12.00	16.30
HCT	32.8	I	%	36.7	47.1
MCV	95.1		fL	80.0	100.0
MCH	32.1		pg	27.0	33.4
MCHC	33.8		g/dL	32.5	36.1
RDW	15.9		%	12.1	16.2
RDW-SD	50.4	H	fL	36.5	46.0
PLT	598.1	Rh	×10^3^/*μ*L	150.0	450.0
MPV	8.79		fL	7.40	11.40

## Data Availability

The data supporting the findings of this study are available within the article.
